# Large Pelvic Hematoma after UroLift® Procedure for Treatment of BPH with Median Lobe

**DOI:** 10.1155/2022/7065865

**Published:** 2022-03-16

**Authors:** Max J. Roehmholdt, Dennis F. Bentley

**Affiliations:** Cleveland Clinic Foundation – Akron General Medical Center, 1 Akron General Avenue, Akron, OH, USA 44307

## Abstract

The UroLift® procedure is a minimally invasive technique used to treat benign prostatic hyperplasia (BPH) in the office or hospital setting. As of 2021, over 200,000 of these procedures have been performed, with an excellent safety profile. We present a case report of a patient who underwent the UroLift® procedure and was found to have a 16.5 cm pelvic hematoma within 16 hours. This study was done as a retrospective chart review. In addition, a comprehensive review of the literature was performed, and all relevant government and company websites were reviewed for thorough evaluation. The patient had an uncomplicated inpatient UroLift® procedure for BPH using 5 implants and was discharged from the hospital without incident. The patient presented to the emergency department with abdominal pain 16 hours after the procedure, and a 16.5 cm pelvic hematoma was found on computerized tomography (CT) scan. Since 2015, there have been 27 cases of pelvic hematoma after UroLift® reported to the United States Food and Drug Administration (FDA), and only 2 cases published in the literature. Our patient required hospital admission for 3 days and 3 units of packed red blood cells, but no surgical exploration or intervention. The procedure was technically successful as it improved the patient's voiding and lower urinary tract symptoms (LUTS) as of 2-month follow-up. Potential etiologies include implant firing depth beyond the extent of the prostate, as well as treatment of the median lobe.

## 1. Introduction

The UroLift® prostatic urethral lift is a minimally invasive surgical technique to treat benign prostatic hyperplasia (BPH) in the office or hospital setting. The advance of pursuing this over the traditional approach of transurethral resection of the prostate is its favorable side effect profile and paucity of complications. As UroLift® is being utilized more frequently in the urologic community, more significant complications are being described. A rarely reported complication of a pelvic hematoma has been described in the literature in the past two years, and we describe another unique case of such, but this time in the setting of the treatment of the median lobe of the prostate, never before described.

## 2. Case Presentation

The patient is a 60-year-old male with a history of BPH with lower urinary tract symptoms (LUTS), managed with Flomax and Proscar once daily each, who wished to be off his BPH medications. He was otherwise healthy and did not take any other medications, including neither anticoagulants nor antiplatelet medications. He was interested in minimally invasive surgical management, including the UroLift® procedure. He then underwent office cystoscopy, which demonstrated a small but mobile median lobe of the prostate. Office transrectal ultrasound of the prostate was performed to determine prostate volume, which was 42 grams. All risks, benefits, and options were discussed, and the patient elected to proceed with the UroLift® procedure.

The patient had a preoperative urine culture, which was negative.

The patient underwent an ostensibly uncomplicated UroLift® procedure in the operating room, under general anesthesia. Intraoperatively, two implants were placed on the right (9 o'clock position between the verumontanum and 2 cm distal to the bladder neck), and three implants were placed on the left (3 o'clock position between the verumontanum and 2 cm distal to the bladder neck), with the third used to specifically address the small albeit mobile and median lobe and tack it to the left side. The implants were placed in the usual standard manner. Final cystoscopy demonstrated a patent anterior urethral channel and no significant bleeding at the implant sites. The patient was able to void spontaneously in the postanesthesia care unit and was discharged home the same day.

**Figure 1:**
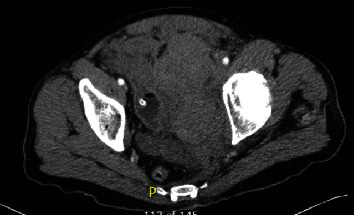
Initial CT abdomen and pelvis in ED (axial): 16 hours postop with large pelvic hematoma.

**Figure 2:**
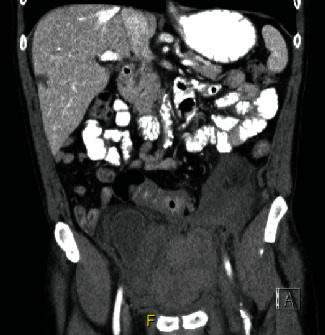
Initial CT abdomen and pelvis in ED (coronal): 16 hours postop with large pelvic hematoma.

On postoperative day #1, 16 hours after the procedure, the patient presented to an emergency department (ED) with abdominal pain. He underwent a computerized tomography (CT) scan of his abdomen and pelvis, and a 14.5 cm by 15 cm by 16.5 cm pelvic extraperitoneal hemorrhage with extension into the left retroperitoneum was found (Figures 1 and 2).

Initial laboratory data at that time included leukocytosis of 26.5, hemoglobin of 13.6, creatinine of 0.89, lactic acid of <0.3, and international normalized ratio (INR) of 1.1. A 16 French Foley catheter was placed for initially pink urine, which turned yellow shortly thereafter in the ED. Blood and urine cultures were sent at that time.

In the ED, the patient was given 1 gram of intravenous (IV) ceftriaxone and 2 liters of IV normal saline fluid. Repeat laboratory values demonstrated leukocytosis of 19.7 and hemoglobin of 11. At that point, he was transferred to a tertiary care hospital for further care.

**Figure 3:**
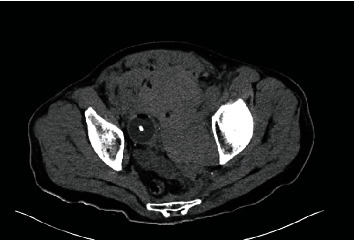
CT pelvis with cystogram at our hospital (axial): displaced bladder; poor cystogram without extravasation.

**Figure 4:**
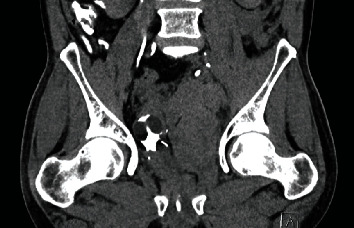
CT pelvis with cystogram at our hospital (coronal): displaced bladder; poor cystogram without extravasation.

Upon admission to that hospital, repeat laboratory data showed leukocytosis increased to 25 and hemoglobin of 9.8. Urology assessed the patient and ordered a CT scan of the pelvis with cystogram, which was negative for extravasation, albeit was a poor cystogram, due to bladder filling limitations as the patient was experiencing severe bladder spasms (Figures 3 and 4).

On postoperative day (POD) #2 (hospital day (HD) #1), daily laboratory data showed leukocytosis improved to 14, hemoglobin of 7.2, and creatinine stable at 0.88. The patient was maintained on scheduled IV ceftriaxone and given 2 units of packed red blood cells. The patient's urine had remained yellow, so he was given a voiding trial and passed. Blood and urine cultures resulted with no growth.

On POD#3 (HD#2), laboratory data showed leukocytosis decreased to 10.9, hemoglobin of 8.2, and creatinine of 0.69.

On POD#4 (HD#3), laboratory data showed leukocytosis had resolved to 7.2, with hemoglobin slightly decreased to 7.5. The patient was on scheduled IV ceftriaxone, blood and urine cultures remained negative, and the decision was made to transfuse the patient 1 additional unit of packed red blood cells—3^rd^ unit of his admission. Repeat hemoglobin was drawn after the transfusion, which was 9.2, and all of his vital signs were stable. The patient was discharged later that day in stable condition, voiding yellow urine.

Outpatient complete blood count on POD#11 demonstrated hemoglobin of 12, and follow-up visit in the office on POD#15 revealed some ecchymosis on the patient's left flank, penis, and scrotum, but the patient otherwise was voiding better and feeling well. Additional follow-up at 2 months postoperatively revealed that the patient's LUTS had resolved.

## 3. Discussion and Literature Review

UroLift® is a device manufactured by NeoTract for prostatic urethral lift, which delivers implants into the prostate to treat prostatic enlargement for the purpose of relieving lower urinary tract symptoms [1].

This was the first complication of its kind seen at our institution since the urologists began performing UroLift® in 2015.

After thorough discussion among the urologists at our institution and reflection on the case with UroLift® representatives, a few ideas were generated as to why such a significant pelvic hematoma may have formed.

One such idea is that the UroLift® device was compressed into the left side too deeply, and when deployed, one of the implants struck a vessel causing the hematoma. Another theory was that when the implant was placed to tack away the median lobe to the left, it may have advanced too proximally or posteriorly in the urethra and, again, struck a vessel, causing the hematoma. The UroLift® device deploys a needle 33 mm deep into the prostatic tissue, which has the potential to fire beyond the extent of the prostate, possibly leading to inadvertent vesicular vascular injuries. The depth can be adjusted based on the amount of compression given into the tissue by the device, prior to firing. The hematoma seen in this case (16.5 cm at its greatest dimension) was a significant size and presented with symptoms 16 hours postoperatively, so it is plausible that a small prostatic or vesicular arterial branch was struck by the implant, rather than a venous injury. Fortunately for the patient, the hematoma was retroperitoneal in nature and only required 3 units of packed red blood cells, without any further operative intervention. A possible solution to this theory could be for NeoTract to provide multiple options for the depth at which the UroLift® implant and needle fires, which could be based on preoperative transrectal ultrasound measurements. Therefore, in patients with smaller prostate glands, a shallower depth implant could be used, which would theoretically provide the same clinical results for relief of lower urinary tract symptoms, without risking deeper vascular injuries.

Interestingly, this unique complication has only been described twice in the literature.

An article published in the Gold Journal of Urology in November 2019 described a case report of a patient undergoing UroLift® and having a postoperative pelvic hematoma [2]. The authors described it as the only known complication of its kind, in the literature [2]. The authors postulated that a short prostatic fossa, high bladder neck, and anterior deployment of the implants (at 2 and 10 o'clock positions) may have contributed to the hematoma in their patient [2].

This article generated some notable commentary on this complication.

One editorial, by Cai et al. from Weill Cornell (NY, NY), described a pelvic hematoma after UroLift® [3]. The patient required 3 units of packed red blood cells, vasopressors, and finally an exploratory laparotomy evacuating 1.5 liters of hematoma including fulguration and oversewing of a bleeding vessel on the left pubis [3]. Their input was that, “It seems that in rare cases, deploying an implant into the extraprostatic space without direct visualization of the final needle path can result in severe complications as described [3].”

Another editorial came from two groups: Tan et al. from the National University Hospital (Singapore) and Gange and Mueller from Summit Urology (Salt Lake City, UT) [4]. They sourced data from the United States Food and Drug Administration's (FDA) Manufacturer and User Facility Device Experience (MAUDE) [4]. Within this database, there was found to be 18 reports of pelvic hematoma since the UroLift® device was introduced [4]. Of these 18 patients, 14 required blood transfusions, 4 required prostatic artery embolization, and 3 required surgery: 1 of those was an open intervention (Cornell editorial), 2 died, and 1 required percutaneous nephrostomy tubes and hemodialysis [4]. Their input included describing how 175,000 patients were treated with UroLift®, which makes a 0.009% estimated pelvic hematoma occurrence [4]. They postulated that the number and percentage was likely lower than in reality, so they encouraged all urologists to report any complications to the MAUDE surveys [4].

A recent article from the University of Buffalo reported a case of pelvic hematoma after UroLift® causing a progression of the patient's chronic kidney disease, requiring 10 units of packed red blood cells and initiation of dialysis [5].

Currently, on the FDA's website for UroLift® MAUDE, since 2015, there have been a total of 27 reports of the adverse event of pelvic hematoma after UroLift® [6]. As of 2021, NeoTract/Teleflex reported that there have been over 200,000 patients treated with UroLift®, culminating in a reported 0.0135% incidence of hematoma.

We hypothesize that since these hematomas formed and were found after UroLift®, then there is a significant possibility that clinically insignificant hematomas also form but never cause enough of an issue to the patient to require hospital visits or imaging studies to discover them. In addition, contributing to this database requires urologists to submit their adverse events, which requires knowledge of where to submit them, as well as being time-consuming. Perhaps, hematoma after UroLift® is more common than is known.

Further studies could be performed to assess patients treated with UroLift®, who have median lobes of the prostate and then determine if there is a higher rate of pelvic hematoma.

According to the MedLift study, there is good data to support the use of UroLift® in patients even with a small median lobe, but there is a possibility that in addressing the median lobe, there is a higher chance of striking a vessel with the implant, causing a hematoma [7]. Therefore, we advise additional caution be taken when using the UroLift® device on a patient with a median lobe of the prostate.

We present a rare complication of a large pelvic hematoma after a minimally invasive BPH procedure, the UroLift®. Potential etiologies include implant firing depth beyond the extent of the prostate, as well as treatment of the median lobe. We encourage all urologists to report any complications of the UroLift®, to ensure there is an accurate representation of the risks of the procedure, and to bring more attention to this potential complication.

## Data Availability

Readers can access data supporting the conclusions of the study by accessing the FDA's MAUDE website and through further research studies regarding pelvic hematoma after UroLift® on a patient with a median lobe.
